# Clinical Study of Using Biometrics to Identify Patient and Procedure

**DOI:** 10.3389/fonc.2020.586232

**Published:** 2020-12-01

**Authors:** Jason W. Sohn, Haksoo Kim, Samuel B. Park, Soyoung Lee, James I. Monroe, Thomas B. Malone, Timothy Kinsella, Min Yao, Charles Kunos, Simon S. Lo, Robert Shenk, Mitchell Machtay

**Affiliations:** ^1^ Radiation Oncology, Allegheny Health Network, Pittsburgh, PA, United States; ^2^ Proton Therapy Center, National Cancer Center, Goyang, South Korea; ^3^ Cure-In Incorporated, Goyang, South Korea; ^4^ Radiation Oncology, Mercy Hospital South, St. Louis, MO, United States; ^5^ Carlow International Incorporated, Fairfax, VA, United States; ^6^ Radiation Oncology, Warren Alpert Medical School of Brown University, Providence, RI, United States; ^7^ Radiation Oncology, University Hospitals of Cleveland, Case Western Reserve University School of Medicine, Cleveland, OH, United States; ^8^ Cancer Therapy Evaluation Program, National Cancer Institute, Rockville, MD, United States; ^9^ Radiation Oncology, University of Washington School of Medicine, Seattle, WA, United States; ^10^ Surgical Oncology, University Hospitals of Cleveland, Case Western Reserve University School of Medicine, Cleveland, OH, United States; ^11^ Penn State Cancer Institute, Hershey, PA, United States

**Keywords:** biometrics, assurance, surgery, oncology, verification

## Abstract

**Purpose:**

To reduce patient and procedure identification errors by human interactions in radiotherapy delivery and surgery, a Biometric Automated Patient and Procedure Identification System (BAPPIS) was developed. BAPPIS is a patient identification and treatment procedure verification system using fingerprints.

**Methods:**

The system was developed using C++, the Microsoft Foundation Class Library, the Oracle database system, and a fingerprint scanner. To register a patient, the BAPPIS system requires three steps: capturing a photograph using a web camera for photo identification, taking at least two fingerprints, and recording other specific patient information including name, date of birth, allergies, *etc*. To identify a patient, the BAPPIS reads a fingerprint, identifies the patient, verifies with a second fingerprint to confirm when multiple patients have same fingerprint features, and connects to the patient’s record in electronic medical record (EMR) systems. To validate the system, 143 and 21 patients ranging from 36 to 98 years of ages were recruited from radiotherapy and breast surgery, respectively. The registration process for surgery patients includes an additional module, which has a 3D patient model. A surgeon could mark ‘O’ on the model and save a snap shot of patient in the preparation room. In the surgery room, a webcam displayed the patient’s real-time image next to the 3D model. This may prevent a possible surgical mistake.

**Results:**

1,271 (96.9%) of 1,311 fingerprints were verified by BAPPIS using patients’ 2^nd^ fingerprints from 143 patients as the system designed. A false positive recognition was not reported. The 96.9% completion ratio is because the operator did not verify with another fingerprint after identifying the first fingerprint. The reason may be due to lack of training at the beginning of the study.

**Conclusion:**

We successfully demonstrated the use of BAPPIS to correctly identify and recall patient’s record in EMR. BAPPIS may significantly reduce errors by limiting the number of non-automated steps.

## Introduction

Patient identification and procedure verification are well-known problems in the medical industry. The number of serious or even fatal consequences is growing. Accreditation by the Joint Commission demands accuracy of patient identification by using at least two patient identifiers (1).

Unfortunately, the most popular identifier, wristbands, is proving to have an unacceptable error rate. These misidentifications can lead to a medical misadministration and falls under the category of a ‘sentinel event’. A ‘sentinel event’ is defined by the Joint Commission as “an unexpected occurrence involving death or serious physical or psychological injury, or the risk thereof ” (2). The sentinel report update in December 2001 from the Joint Commission analyzed 126 incidents for root causes and determined 76% involved surgery on the wrong body part or site; 13% involved surgery on the wrong patient; and 11% involved the wrong surgical procedure. Of the 126 incidents, only 81% were self-reported (3). The Joint Commission goes on to point out that wrong site surgery data collected by other organizations suggested a significant amount of under-reporting to the Joint Commission.

**Figure 1:**
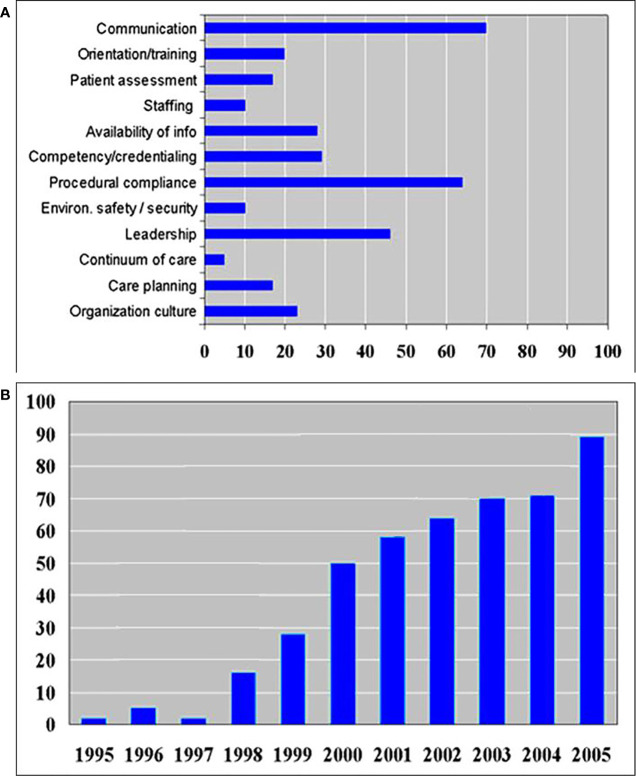
**(A)** Number of various root causes of wrong site surgery in 2005 as reported by the Joint Commission (4). **(B)** Sentinel event trends’ report showing the number of wrong surgeries increases every year (4).

The Joint Commission analyzed the causes of misadministration in 2005 as illustrated in **Figure 1A** (4). The lack of communication, patient assessment, and availability of information caused 113 ‘wrong surgeries’. The number of wrong surgeries still increases every year as shown in **Figure 1B** (4).

The ‘two patient identifiers’ specified by the Joint Commission have a two-fold purpose: firstly, to reliably identify the individual and secondly, to match the service or treatment to that individual. The identifiers may be in the same location, such as a wristband. They must be directly associated with the individual, and the same two identifiers must be directly associated with the treatments or procedure. The ‘two-identifier’ requirement also applies to an ‘order for care’ and to report critical test results. Wristband systems are the most common patient identifier in use. These identifiers play an important role in the Joint Commission protocol to reduce surgical misadministration.

Effective July 1, 2004, compliance with the Universal Protocol for Preventing Wrong Site, Wrong Procedure, Wrong Person Surgery has been required of all Joint Commission accredited organizations, to the extent that these requirements are relevant to the services provided by the organization (5). An important concept is the “time out” quality check now used in procedures. In addition to patient, procedure, and site verification, the “time out” must include verification of correct patient position and availability of correct implants and any necessary special equipment.

There are a few competing technologies for patient identification, namely barcode and radio frequency identification (RFID) chips. RFID can be used to track the patient’s location and extract patient information using a remote scanner (6–8). However, RFID has a weakness in security since it can be read with an illegitimate remote scanner. This issue has been reported by experts (9, 10). Facial recognition system also has been introduced and used in commercial systems.

Barcode or RFID chips can be taped on patient wristbands. However, their effectiveness of identifying patients is not convincing. There are two critical studies of the barcoded wristbands, which have a significant pool. The first study was reported by the State University of New York, Downstate Medical Center in Brooklyn, New York (11). The wristband error rates were tracked over a two-year period.

During the two years, wristbands were examined 1,757,730 times, and 45,197 wristband errors were found. The mean wristband error rate for the first quarter was 7.4%. However, by the eighth quarter, the mean wristband error rate had fallen to 3.05%. Even with this improvement, sentinel events continued to rise as shown in **Figure 1B**.

A second study was conducted at the Veterans Affairs Medical Center in West Los Angeles, California, and compared wristband identification errors for 712 hospitals. Phlebotomists checked 2,463,727 patients’ wristbands, finding 67,289 errors (11).

Ten percent of these hospitals had error rates of 10.9% greater. Patients’ wristbands were missing in 33,308 cases, representing 49.5% of errors. Multiple wristbands with different information occurred 8.3% of the time; wristbands with incomplete data 7.5%; erroneous data 8.6%; illegible data 5.7%; and patients wearing wristbands with another patient’s identifying information occurred 0.5% of the time.

We developed a biometric system using fingerprints to meet the Joint Commission’s recommendations and minimize error rates. Our system was designed to interact with Radiology Information System (RIS) and EMR and can potentially eliminate most of the problems associated with wristband systems while helping clinics meet the goals set by the Joint Commission. Patient misidentification is limited to the failure rate of fingerprint identification, which is approximately one out of a billion (provided the patient can offer two fingerprints). Also, procedure verification can be performed biometrically by interacting with the relevant patient database such as an electronic chart or RIS system. Moreover, fingerprints are not subject to loss, damage, or switching between patients in the same way as plastic wristbands. Multiple records for one patient can be prevented since there is one set of unique biometric information (12, 13). Also, patient privacy is maintained, particularly for outpatients who wish to keep their status private by not wearing wristbands. This system can be used to identify and provide patient’s vital information to clinicians when a patient is not able to provide his/her information. Patients who are suffering from Alzheimer’s, unconsciousness, impaired hearing, or language difficulty can potentially benefit from this technology. Sixthly, our system can be used with other biometric systems (such as retinal or face scanners) by integrating their drivers and pattern recognition algorithms.

The project team included a Human Factors Analysis expert to insure the total system (hardware and humans) functions, not just the technical aspects. System design has recently begun to focus on human factors’ considerations. According to Kukula et al. (14), The Human-Biometric Sensor Interaction (HBSI) is a conceptual model that is centered on the relationship between the human providing the biometric characteristic, such as a fingerprint, to the biometric sensor, a relationship that becomes more critical to understand and research as biometric sensors become pervasive in society (14).

The successful deployment of biometric systems, regardless of application, needs to take into consideration how individuals interact with the device. Failure to do so will cause a degradation of the optimal performance of the biometric sensor, causing problems such as failure to acquire, failure to enroll, and impacts on the false rejection rate. Moreover, if the individual cannot successfully interact with the biometric device, there is a potential for a failure to function even when the device is implemented.

While the human in the Human-Biometric Sensor Interaction construct is the person being identified (*e.g.* the patient), the other equally important person in the interaction is the user, the person using the fingerprint biometric device to identify the patient. In fact, the success or failure of the biometric device usually depends on the performance of the user who will provide instructions to the patient and guide the patient through correct finger orientation, force application, and placement in order to acquire a fingerprint sample of sufficient quality for reliable identification. Rood and Jain (15) and Kukula et al. (14) pointed out the complexity of designing a biometric system which is based on three main attributes—accuracy, scale (size of the database), and usability (15).

## Methods and Materials

### System Design

Our patient identification system uses optical fingerprint scanners for biometric data collection, creates a fingerprint database, and interacts with the established clinical patient databases. For the creation of an identification database, our program captures a photograph using a web camera, stores two or more fingerprints, and records brief patient identification information such as names and date of birth. An optional single fingerprint mode is available at the expense of reduced accuracy. During an identification process, our system accepts a fingerprint, identifies the patient, verifies with a second fingerprint, and opens the correct patient record in the R&V database.

### Hardware

**Figure 2:**
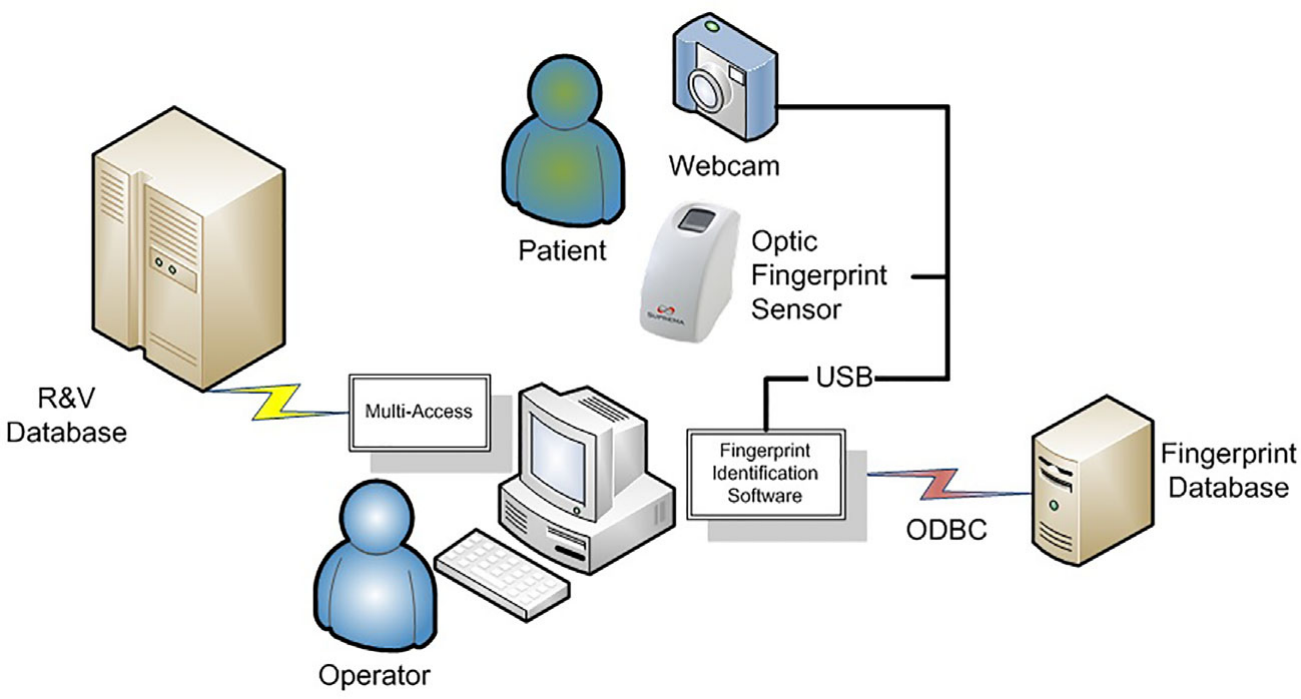
Schematic of the BAPPIS system.

The hardware consists of personal computers, a database server, optical fingerprint scanners, and web cameras. They are connected *via* universal serial bus (USB) cables as illustrated in **Figure 2**. The hospital computers were Pentium 4 personal computers with a speed of 1.6 GHz, utilizing Windows XPTM operating system. The database server was configured with ORACLE database 11G Release.

The model of the optical fingerprint scanner is SFR300-S (Suprema Inc.). It has a 500 dpi/256 Gy scale optical fingerprint sensor in a plastic case. The scan window is 16 mm × 18 mm. Its physical dimension is 40 mm (width) × 77 mm (length) × 70.5 mm (height). Scanning time takes less than a second. The web camera used is an inexpensive CS431 by Intel.

### Software

Our program is written in Microsoft^®^ Visual C++. Each patient’s record in the fingerprint database consists of three components: fingerprint images, a photo image, and a text record. Each scanned fingerprint has key information extracted and stored in a proprietary format (about 368 bytes per scan, stored in 256 bit AES encryption) (16). The photo image is currently in the form of a 173 kB bitmap. Text information storage is trivial and therefore each patient record takes up approximately 175 kB. A 40-GB storage space could store 228,000 patients or 4,000 patients in a typical computer CD (700 MB storage).

Fingerprint recognition is handled through the UniFinger Engine, a proprietary algorithm provided with the scanner. When we use fingerprint recognition engines, we need to take into account two possible errors. The first one is the false match (FM), and the second one is the false non-match (FNM). The FM refers to the mismatching fingerprints. The FNM is the inability to match a fingerprint to one in the database. These two errors can be adjusted as variables in our system. If the FM rate is set low because false match is a serious security problem, then the fingerprint recognition engine rejects even the tiny mismatch. As a result, the rate of false non-match (rejection) increases. This will translate into more frequent rejections and retries. According to the result of the international fingerprint verification competition in 2006 (FVC2006), the fingerprint recognition algorithm that we are using resulted in a 1.36% FNM rate when FM rate was set to 0%, and a 0.79% FNM rate when the FM rate was set to 0.1% (FVC2006, Database #2, images scanned with the optical fingerprint scanner) (17).

We employed two-fingerprint identification step with two different fingers in our system. We set FM rate to 0.1% for the first fingerprint identification to avoid FNM. Then, for the second fingerprint, we verify the identification result by setting FM rate to 0.000001%, so that we can prevent the false positive identification. With two fingerprints, the identification error probability is one out of 100 billion. In single fingerprint mode (using one fingerprint), the error probability is one in 100,000.

We also utilize a quality index of the initial fingerprint registration. UniFinger engine provided the fingerprint image quality index in a percentile scale. A good quality fingerprint has distinguishable patterns and features that allow the extraction of features that are useful for subsequent matching of fingerprint pairs. The fingerprint image quality index is a predictor of a pattern recognition algorithm’s performance (18), and it is considered as a good fingerprint image if it has the quality index above 50 (19). We set the index threshold to 80 for determining a successful fingerprint registration. During our preliminary experiments in the following section, there was no false non-match error when the quality index threshold was set to 80. All fingerprints are stored as long as the quality exceeds the index of 80 for our two-fingerprint identification process. For special cases, we added a single fingerprint identification option.

Our scanner system connects to the fingerprint database using an open-database connectivity (ODBC) driver. ODBC provides a standard method for using database management systems (DBMS). ODBC is designed to be independent of programming languages, database systems, and operating systems (20). ODBC manages this by inserting a middle layer, called a database driver, between an application and the DBMS (21). The purpose of this layer is to translate the application’s data queries into commands that the DBMS understands.

**Figure 3:**
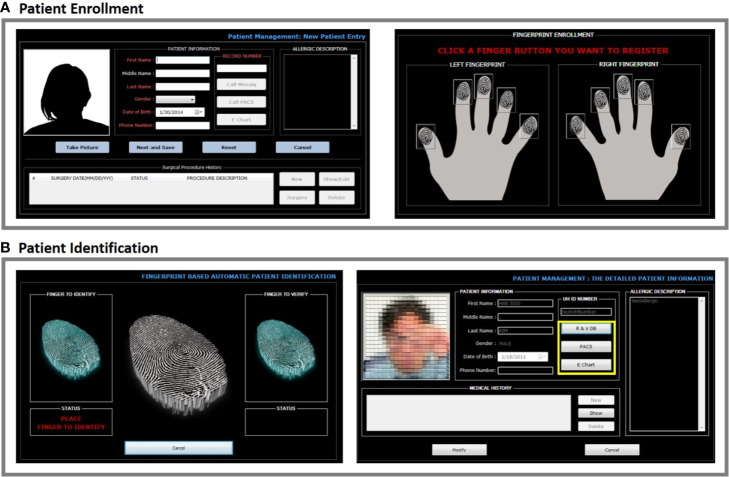
**(A, B)** After a patient is enrolled by entering name, birthday, and hospital ID number, a photo can be directly taken using the BAPPIS. Then, a match mode can be set to either two fingerprints or single fingerprint for identification. Once a mode is selected, finger prints can be taken.

We felt it was important to make the initial record creation of a patient’s one seamless operation run by a single program. To create a new record, the text information is entered and the patient was photographed with a web camera as displayed in **Figure 3A**. Immediately afterward, all fingers were scanned if the quality index exceeds 80 (about 1 s each). One or two fingerprint identification options can be selected in **Figure 3B**. Fingerprints, text information, and the patient photo-image are directly added into the patient record in our identity database. No files are added by importing into a patient record, an operation which can lead to mistakes (*e.g.*, importing a picture to the wrong patient).

After scanning one finger, the system identifies a patient (about 1 s/1,000 records to search), and the patient photograph is displayed. Scanning only one finger is an option. A second finger is then scanned to confirm identification. The photograph display is an additional visual check for the patient and staff.

**Figure 4:**
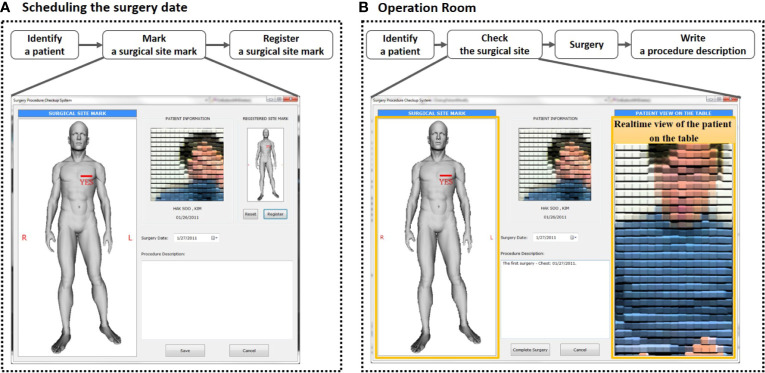
**(A)** At each patient visit, after fingerprint(s) are scanned, the system identifies and displays patient photo. It provides the connection to other hospital databases, such as Mosaiq, PACS, and electronic medical record. **(B)** In the surgical department system, the fingerprint identification system includes surgical procedure verification. The final display of the consultation sketch and the patient on the table provides a clear comparison between the two. Procedure description notes are also displayed for additional confirmation.

We designed the software to interact with other hospital databases using a modular approach. The current module interacts with the radiation oncology Mosaiq (Elekta, Atlanta GA), RIS (PACS), and EMR database as shown in **Figure 4A**. Our main software matches the fingerprints to the patient in the fingerprint database, which then yields the medical record number (MR). The module uses the MR number in Mosaiq database to retrieve the patient file.

Our project included two departments to test our prototype. For the testing of our prototype system and full clinical study, we acquired an Institutional Review Board (IRB) protocol (CC862) approval. A timing routine was programmed into the software to track the amount of time the user interacted with the software as one measure of functionality and usability. The measurements were saved in log files. The measurements included user logins, fingerprint recognition failures, process durations, *etc.* A combined fingerprint database was created from both departments, one database from each department. This was designed to prevent duplicate files for a patient.

All operations with patient information were automatically recorded on encrypted database log files. The files were reviewed once a week by the Principal Investigator. For physical security, the computer database was protected in a locked data server room, while the clinical computers were subject to hospital security measures.

For radiation therapy, our system was set up at a receptionist desk and treatment machine console areas to perform two basic tasks: 1) The identification of a patient and entering them into the treatment queue; and 2) The identification of a patient being prepared for treatment and the loading of their treatment settings to the machine. At the receptionist desk, the hardware includes a webcam with a resolution of 640 × 480, a personal computer, and an optical fingerprint scanner. A receptionist enrolled new patients through the process. When patients returned to the clinic for daily scheduled treatments, they were asked to scan one/two fingers depending on whether the single or two-fingerprint identification mode was being used on the scanner at the reception desk. Our program identified these patients and added them to the waiting list maintained in the Mosaiq system. This process allowed the therapists at the treatment machine to know that the patient had arrived for treatment.

When a patient walked into the treatment room, he/she was asked to scan his/her fingers. The photo window was displayed for the therapist to use for visual confirmation of the patient as a second identification after the patient’s fingerprint match. Next, our system retrieved the patient treatment machine settings in the Mosaiq system for their radiotherapy.

At each patient visit, after fingerprint(s) were scanned, the system identified and displayed the patient’s photo. It provided the connection to other hospital databases, such as Mosaiq, RIS, and electronic medical record.

We installed the BAPPIS system in the UH Cleveland Medical Center Surgery Department where an average of 40 breast surgeries per month are performed in 30 surgical suites. Our systems were installed in two breast consultation suites, one surgery preparation room, one surgery reception desk, and two consultation rooms. Our software provided a module for the surgery department, which allowed a surgeon to create a procedure/orientation verification display consisting of an anatomical sketch, procedure description, and digital image of the patient in treatment position. Once a patient information was recorded in the system, his/her surgeon could create a short surgery description and added an anatomical sketch appropriate to the surgical site from a library, which was available from our software. Most of the information was entered by the surgeon in the consultation room, allowing them to discuss the procedures with the patient. The surgeon clearly marked “yes” on the sketch following the Joint Commission recommendation (5) for marking the surgical area on the patient’s skin. The digital image of the patient in treatment position with the surgeon’s marks on the skin was taken in the surgery prep room. This display was available in the operating room for the “time out.”

At the time of surgery, as the patient entered the surgical suite, his/her fingers were scanned and identified. Once the patient was placed on the surgical couch, a photo was taken and displayed next to the anatomy sketch as illustrated in **Figure 4B**, which could be displayed side-by-side on the same monitor. During “time-out,” this screen helped to identify the patient, verify the surgical procedure, and confirm orientation.

Once the surgery was completed, the patient file including notes and digital image after surgery was exported to an electronic patient chart and saved in our software system. This eliminated possible confusion during future additional surgeries.

A module was designed for adding an authorized user along with their fingerprint to the database. There was a range of privileges that could be assigned to the user based on security requirements and the users’ function. Only privileged users were able to open a patient’s history and modify the patient’s information.

### Fingerprint Servers

We created two clinical fingerprint databases, one for the radiation oncology department and one for the surgery department. The two clinical databases were combined and stored in a node server. This multi-database system could be scaled up to model an entire hospital with multiple satellite clinics.

**Figure 5:**
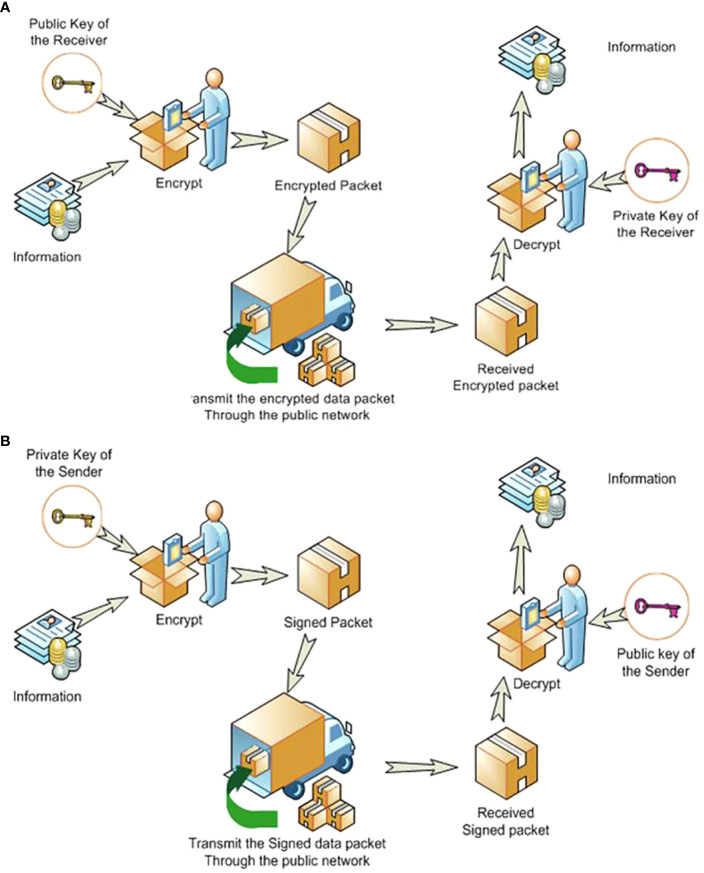
The asymmetric cryptographic system uses two keys: a public key known to everyone and a private or secret key known only to the recipient of the message. Sending messages goes through two processes—**(A)** Encrypting the information for secure transmission. **(B)** Providing a digital signature for certifying the sender.

The multi-database architecture used asymmetric cryptography for safe data transfer when communicating between databases. A pair of keys was used to encrypt and decrypt a message and signature so that it was transmitted securely. The communication between fingerprint database servers was therefore confidential and secure, as required by HIPAA (22, 23). Our proposed asymmetric cryptography is summarized in **Figure 5** (24).

Asymmetric cryptography can be illustrated by an example where ‘John’ wanted to send a fingerprint to ‘Susie’ securely. A fingerprint was sent to Susie (the remote server) encrypted using Susie’s public-key, and John’s signature (credibility) was included, but encrypted with John’s private-key. John could get Susie’s public key from a public administrator. When Susie got the fingerprint, she decrypted it with her private key, to which no one else should have access. However, she had to verify that it was John who sent the package. John sent his signature encrypted with his private key to Susie who used John’s public key to decrypt his signature. This asymmetric cryptography had been used for internet banking (25).

We also implemented the Common Biometric Exchange Formats Foundation (CBEFF) proposed by the National Institute of Standards and Technology (NIST) (26). CBEFF was originally developed for the biometric authentication *via* network as it dictates the header information to be used for standardized communication when transmitting biometric information (like fingerprints).

## Results

**Figure 6:**
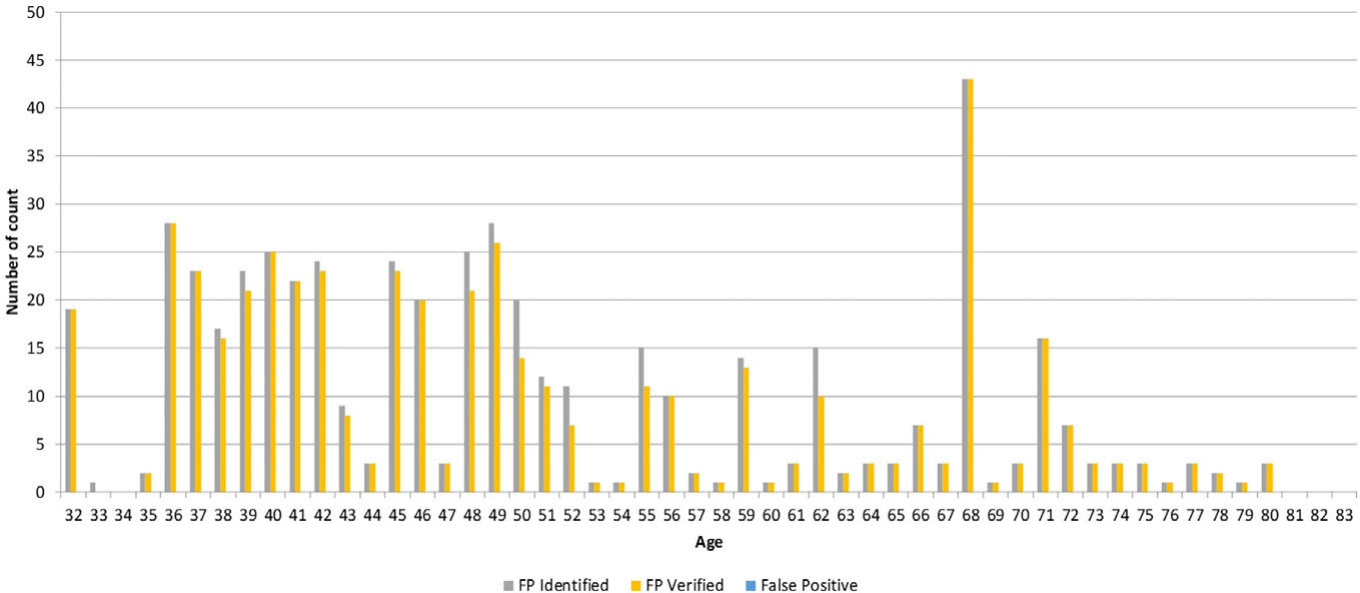
Graph demonstrating the number of identified, verified, and false positive fingerprints for each patient.

BAPPIS identified 1,311 fingerprints from 143 patients prior to treatment as a graph created in **Figure 6**. 1,271 fingerprints were verified with the system. The fidelity rate for BAPPIS was 96.9% for identified fingerprints. False positive recognition was not reported. The rate was not 100% because the operator did not verify the second finger after identifying the first fingerprint. This could be attributed to the lack of training at the beginning of the study and increased workload of the therapists who had to use BAPPIS in addition to the established identification method. Any false positive identification required the receptionist or therapists to page the investigator(s) of the study currently assigned to monitor the clinical trial. A full record of the event would be recorded in a log for analysis. Technical problems were automatically recorded in a log file. Note that any problems with the identification system did not interfere with normal clinical operations due to its auxiliary nature. Patient records and treatments were always available using the standard clinical operation procedures.

## Discussion

We successfully demonstrated the use of BAPPIS to correctly identify and recall patient plan information using fingerprint identification. BAPPIS could help significantly reduce the number of errors by limiting the number of non-automated steps in the treatment delivery process. The cost of this system is very minimal. A fingerprint scanner’s cost was less than $100, and data server was about $5,000.

A significant effort was made toward developing a user-friendly interface (usable, reliable, readable, intuitive in terms of meeting user expectations, aligned with used procedures, easy to train, easy to retain training, and easy to determine the next step in the procedure). And emphasis was placed on creating a system that patients and professionals could easily use with minimal training. We performed a human factor analysis in every interface during an identification and minimized challenges to the successful operation of the device, such as when handling patients with cognitive or attention limitations, frail elderly patients with loose skin in the fingertips, and patients with vision, hearing, and language difficulties. Every attempt was made to include persons having these characteristics. The inclusion of these patients as test subjects was not subjected to sampling restrictions associated with statistical predictive validity since the total number of subjects was restricted for practical purposes, and the impetus for the testing was not to predict levels of performance in the general population, but rather to identify and eliminate performance problems associated with the device.

We opened an IRB protocol and received all participated patients’ consents. We followed HIPAA regulations and recommendations for medical information protection. Our system only stored the identification information (no medical chart information) with data encryption. Firewall software was installed on the database server, and security patches were monitored and installed. The patient database only exists within the secure hospital intranet and was never exposed to the internet. Communication between hospitals was through virtual private networks (VPNs). There were few patients who had concerns about taking fingerprints and did not enroll.

The comparison between current identification and the use of BAPPIS will be done in the future. Since clinical studies with an IRB required the use of the current identification method by asking name and birthday in addition to BAPPIS, the error rates between two methods were not analyzed.

Since we initiated our study, many other biometric systems have been introduced such as fingerprint and vein scanners, palm scanners, and face recognition systems.

## Conclusions

We successfully demonstrated the use of BAPPIS to correctly identify and recall patient’s record in EMR. BAPPIS may significantly reduce errors by limiting the number of non-automated steps.

## Data Availability Statement

The original contributions presented in the study are included in the article/supplementary materials; further inquiries can be directed to the corresponding authors.

## Ethics Statement

The studies involving human participants were reviewed and approved by Case Western Reserve University and University hospital IRB. The patients/participants provided their written informed consent to participate in this study.

## Author Contributions

JS took charge in the PI and writing of the manuscript. HK and SP developed the system as Co-Inv and wrote the manuscript. SL contributed to the writing of the manuscript. JM and TM were in charge of Co-Inv and writing of the manuscript. TK, MY, CK, SL, RS, and MM participated in the research and writing of the manuscript. All authors contributed to the article and approved the submitted version.

## Funding

The project in this manuscript was supported by an AHRQ 5R18HS017424 GRANT. For this clinical study, an IRB was approved by the University Hospitals of Cleveland and Case Western Reserve University School of Medicine. Case Comprehensive Cancer Center; 5P30CA043703.

## Conflict of Interest

SP was employed by Cure-In Incorporated. TM was employed by Carlow International Incorporated.

The remaining authors declare that the research was conducted in the absence of any commercial or financial relationships that could be construed as a potential conflict of interest.
